# Short Peptides as Excipients in Parenteral Protein Formulations: A Mini Review

**DOI:** 10.3390/pharmaceutics17101328

**Published:** 2025-10-13

**Authors:** Dorian Migoń, Zbigniew Jaremicz, Wojciech Kamysz

**Affiliations:** Department of Inorganic Chemistry, Faculty of Pharmacy, Medical University of Gdańsk, Hallera 107, 80-416 Gdańsk, Poland

**Keywords:** protein formulation, protein aggregation, protein drug product development

## Abstract

Biopharmaceutical medicines represent one of the most dynamic sectors of the pharmaceutical industry, with therapeutic proteins forming the largest and most important group. Their structural complexity and inherent sensitivity to chemical and physical stressors, however, continue to pose major challenges for formulation development and long-term stability. Short peptides have emerged as a promising yet underutilized class of excipients for protein-based drug products. Their modular architecture allows for precise tuning of physicochemical properties such as polarity, charge distribution, and hydrogen-bonding potential, thereby offering advantages over single amino acids. Experimental studies indicate that short peptides can serve multiple functions: stabilizers, antioxidants, viscosity-lowering agents, and as lyo/cryoprotectants or bulking agents in lyophilized formulations. Notably, the relatively small and chemically defined space of short peptides—approximately 400 possible dipeptides and 8000 tripeptides—makes them particularly amenable to systematic screening and computational modeling. This enables rational identification of candidates with tailored excipient functions. This review summarizes current knowledge on the use of short peptides as excipients in parenteral protein formulations, with a focus on their functional versatility and potential for rational design in future development.

## 1. Introduction

Biopharmaceutical medicines constitute a significant, fast-growing sector of the pharmaceutical industry [1]. Specifically, this group of modern and dynamically developing therapeutics accounted for half of the best-selling drugs of 2020 [2]. Furthermore, the loss of patent protection and exclusivity rights of many biological medicines allows biosimilar medicines to enter the market. Hence, a significant interest in the production of cheaper, off-patent counterparts to already approved biological medicines has been observed among pharmaceutical companies. As a result of health care cost reduction, the biosimilar market will provide a substantial contribution to an increase in access to innovative therapies for patients [3]. One of the largest groups of biologicals is therapeutic proteins. Development of drug products based on the abovementioned molecules is challenging, because proteins, due to their structural complexity, are characterized by an enhanced susceptibility to various environmental factors [4,5].

Nearly all commercially available protein-based drugs are formulated as solutions or lyophilizates for parenteral administration [6]. Their degradation can occur during manufacturing, processing, storage, and administration to the patient. This is common in both liquid and solid preparations. The extent of degradation depends on factors such as the amino acid sequence, isoelectric point, and hydrophobicity of the molecule, as well as storage temperature, primary packaging, and the composition of the medicinal product. Degradation products may reduce the drug activity and, in the worst case, increase its immunogenicity. Therefore, the primary goal of formulation (i.e., a carefully selected combination of excipients) is to ensure protein stability so that the medicinal product remains effective and safe for patients throughout its entire intended shelf-life.

As mentioned above, many degradation pathways are strongly dependent on the pH and ionic strength (composition) of the solution. While it is nearly impossible to completely prevent all degradation routes, selecting appropriate excipients can minimize them to an acceptable level [7,8].

Up to date, several review articles regarding excipients utilized in therapeutic protein formulations have been published. As an example, Kamerzell et al. comprehensively compiled excipient categories, protein–excipient interaction mechanisms, as well as biophysical methods utilized to study them [9]. Another interesting example is an article written by Strickley et al., where authors reviewed formulations of commercially available antibodies [10]. Lastly, Bjelošević et al. defined the most important groups of biopharmaceutical excipients utilized in lyophilized presentations [11]. Because our article is mainly focused on latest achievements in excipient group of short peptides, general information on parenteral protein-related excipients was reduced to basic concepts. In order to acquaint oneself with those topics in a more detailed manner, reference to aforementioned publications is advised. A concise overview of excipient categories, classified according to their functional roles in the development and stabilization of parenteral protein therapeutics, is provided in Table 1.

**Figure 1:**
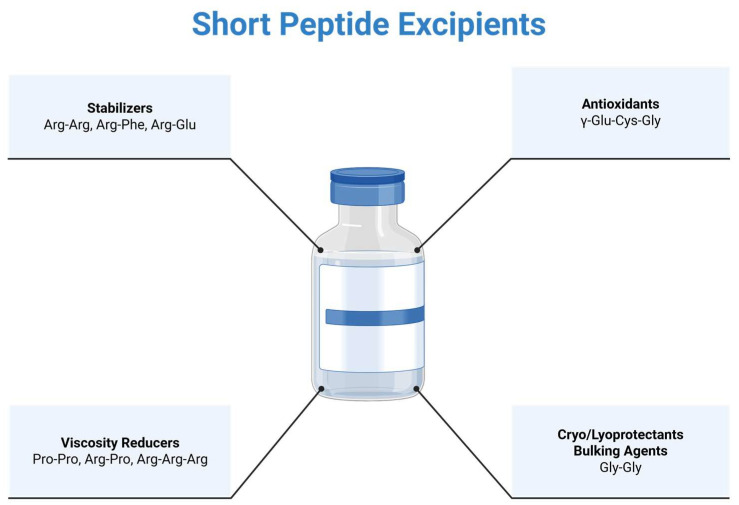
Examples of short peptides explored as excipients in protein formulations, illustrating their potential functional roles.

Short peptides, particularly dipeptides and tripeptides, have emerged as a promising but underutilized class of excipients for parenteral protein formulations. Their modular structure allows for the precise tuning of physicochemical properties such as polarity, charge distribution, and hydrogen bonding capacity, offering more formulation flexibility than individual amino acids. As an example, short peptides have shown potential to act as stabilizers, viscosity reducers, antioxidants, cryoprotectants, and bulking agents in both liquid and lyophilized protein formulations (Figure 1). Moreover, the relatively small and chemically defined space of di- and tripeptides (~400 and ~8000 possible combinations, respectively) makes them amenable to systematic experimental screening or computational modeling strategies aimed at identifying peptide-based excipients with tailored functional profiles [13]. Together, these attributes position short peptides as a versatile and rationally designable platform for next-generation excipient development in protein drug formulations.

This review focuses on the latest achievements in the application of short peptides as excipients in parenteral protein formulations, highlighting their functional roles, mechanisms of action, and formulation contexts—both in solution and in lyophilized products. A particular emphasis is placed on their potential as a rationally designable excipient class for future formulation development.

## 2. Stabilizers

Stabilizers are a class of excipients that encompasses a broad range of molecules aimed at enhancing both the conformational and colloidal stability of proteins. This category includes sugars (primarily sucrose and trehalose), polyols (mainly sorbitol and mannitol), and amino acids (notably arginine and glycine). Although stabilizers represent a chemically diverse group of excipients, each of these compound classes has a long-standing history of use in protein-based drug products. Their concentrations typically range from moderate (0.1 M) to high (1 M) levels. The precise mechanism of action of stabilizing excipients depends on the molecular structure of the excipient, the properties of the target protein, and the formulation context—whether as a liquid, frozen, or lyophilized product. Nevertheless, their effects on protein stability in solution are primarily governed by the interplay between excipient, protein, and water molecules [14,15].

The preferential interaction theory is considered the primary and universal mechanism explaining the stabilizing properties of excipients in the stabilizer class. In this framework, two types of excipient–protein interactions are distinguished. In the first case, the local molality of the excipient at the protein surface is lower compared to the excipient concentration in the bulk solution. This interaction mode, known as preferential exclusion, creates a thermodynamically unfavorable situation for direct excipient–protein contact. The exclusion of excipient molecules from the protein surface and the resulting preferential hydration increase the chemical potential of the protein, which in turn favors the compact, native protein state. Since the surface area of the native state is smaller than that of the denatured state, the equilibrium shifts toward the native form, leading to its stabilization. The extent of preferential exclusion and the corresponding rise in protein chemical potential scale with solvent-exposed surface area, which explains why this effect favors the compact native structure. This mechanism is typically attributed to sugars and polyols [14,15,16,17]. On the other hand, in preferential binding, excipient molecules are enriched at the protein surface relative to the bulk concentration. Arginine is an example of a molecule associated with this type of interaction, as it is believed to have affinity for the side chains of certain amino acids, particularly aromatic residues. Unlike sugars and polyols, which act mainly through conformational stabilization, arginine primarily enhances colloidal stability of proteins. Li et al. proposed that arginine interacts with hydrophobic protein groups to form a cage-like network, thereby reducing attractive protein–protein interactions. This effect not only increases protein solubility but also contributes to viscosity reduction in concentrated formulations [18]. However, in some instances, arginine has been reported to promote aggregation of acidic proteins under low ionic strength conditions, which is attributed to its binding to the protein and neutralizing negatively charged groups [19,20].

Amino acid stabilizers often require moderate-to-high concentrations (≥0.1 M) to achieve beneficial effects, which can limit their use in therapeutic liquid protein formulations due to osmolality constraints. This has driven interest in identifying novel stabilizers with enhanced properties, with short peptides emerging as promising candidates owing to their low toxicity. Among these, arginine-containing peptides are the most extensively studied in protein formulation research [21]. Peptides, in general, offer considerable potential as excipients because of their structural diversity, which may allow for improved selectivity toward aggregation-prone regions of therapeutic proteins.

Early publications on short peptides as additives in protein solutions were primarily framed in the context of understanding the molecular mechanisms of protein aggregation rather than optimizing the formulation of protein therapeutics. These studies often employed di- and tripeptides as model additives to probe the role of specific chemical functionalities—such as hydrophobic side chains, charged residues, or hydrogen-bonding motifs—in modulating intermolecular interactions. As an example, La Rosa et al. investigated the interaction of cytochrome c with Phe and Gly amino acids, and the dipeptides Phe-Gly, Gly-Phe and Ala-His using 1H NMR, MD simulations, and DSC. The authors demonstrated that only Phe-Gly prevented thermal aggregation of cytochrome c at neutral pH, interacting specifically with Gly45 and Phe46 in a sequence-dependent manner [22]. On the other hand, Artemova et al., using a series of biophysical techniques including dynamic light scattering, turbidimetry, fluorescence spectroscopy, and electron microscopy, demonstrated that certain short amphiphilic peptides—particularly Arg-Phe, Ala-Phe-Lys, and Asp-Phe—can accelerate alcohol dehydrogenase and α-lactalbumin aggregation under mild or physiologically relevant conditions. It should be noted that the peptide concentrations used in these studies were relatively low (0.5–4.5 mM), and in some cases—such as Arg-Phe with alcohol dehydrogenase—increasing the concentration or temperature reduced the aggregation-promoting effect [23,24,25].

Expanding on these early insights, arginine-containing peptides have become a major focus, tested extensively across different protein systems. Nuhu et al. demonstrated that Arg-Arg suppressed insulin aggregation at acidic pH but promoted self-association at neutral pH. Contrarily, Arg-Phe almost completely prevented thermal aggregation at pH 7.5 up to 90 °C. [26]. Similarly, Vekaria et al. observed that Arg-Arg increased aggregation of negatively charged proteins (rHSA, ovalbumin), likely via charge neutralization, whereas Glu-Glu stabilized positively charged α-chymotrypsinogen A [27]. Shukla et al. further explored the role of polyarginines (n = 1–4) in different counterion forms, showing that chloride salts destabilized proteins at higher peptide concentration, while sulfate salts consistently improved stability due to strong guanidinium–sulfate pairing. These findings highlight both the promise and complexity of cationic peptides as excipients, where charge balance and counterion identity play decisive roles [28]. Beyond these experimental studies, computational approaches have also been leveraged to identify promising short peptide excipients. Tosstorff et al. used molecular dynamics (MD) simulations to rationalize the stabilizing mechanism of Gly-D-Asn, which markedly improved interferon-α2a stability at low concentrations by interacting with aggregation-prone regions. Notably, Gly-D-Asn outperformed its enantiomer and conventional excipients such as L-arginine and trehalose [29,30]. In another example, Lui et al. employed MARTINI coarse-grained MD simulations to pre-screen hydrophilic dipeptides for their ability to modulate antibody–antibody interactions. Experimental validation confirmed that charged dipeptides, particularly His-His, reduced self-association in good agreement with calculated interaction energies [31]. Finally, patent literature provides additional evidence for the stabilizing potential of short peptides. Somers et al. described multiple di- and tripeptides—including Gly-Gly, Gly-Gly-Gly, Gly-Tyr, and Gly-Phe—as effective stabilizers of erythropoietin omega under both accelerated and stress conditions. In these formulations, peptide excipients either matched or surpassed the protective effects of human serum albumin, highlighting their feasibility as functional excipients in commercial formulations [32]. Together, these studies illustrate that the functional impact of short peptides varies strongly with the protein system and experimental context. Depending on their sequence, charge distribution, and concentration, peptides can either suppress or promote aggregation. Additionally, arginine-based peptides, in particular, show pronounced sensitivity to pH and counterion identity. Short peptide excipients utilized in abovementioned studies are presented in Table 2.

## 3. Antioxidants

Amino acid residues found in proteins, such as His, Met, Cys, Tyr, and Trp, are susceptible to oxidation by reactive oxygen species (ROS). This reaction can occur during production, purification, formulation, or storage of the drug. Trace impurities, such as metal ions and peroxides, may be present in excipients and can, in turn, lead to chemical degradation of proteins. For example, polysorbates 20 and 80 can undergo auto-oxidation, producing hydrogen peroxide [33]. Oxidation in protein drugs should be minimized by optimizing the manufacturing process. Additionally, the use of antioxidant excipients is another approach to protect drugs from oxidative reactions. Three groups of antioxidants are distinguished: (i) true antioxidants; (ii) reducing agents; and (iii) antioxidant synergists [34]. True antioxidants are substances that can react with free radicals, forming intermediate products that terminate oxidation by inhibiting the chain propagation step of free radical reactions. Reducing agents are substances that can react with oxygen via preferential oxidation, thereby removing the source of oxidation; however, they may be consumed during the product’s shelf life (e.g., ascorbic acid or methionine). Antioxidant synergists are compounds that exhibit antioxidant properties indirectly, including chelating agents that bind to metal ions which catalyze oxidation reactions (e.g., EDTA or DTPA). It should be emphasized that the choice of an appropriate antioxidant depends on the structure of the protein drug. For instance, ascorbic acid has been observed to promote methionine oxidation in small peptides and in recombinant human ciliary neurotrophic factor [35]. The most commonly used antioxidants in monoclonal antibody medicinal products include methionine, EDTA, and DTPA. Methionine is present in 10 liquid formulations, all with histidine buffer; EDTA is found in 1 lyophilized product and 8 liquid formulations; while DTPA is used in 4 liquid formulations [10].

Peptide-based antioxidants in parenteral protein formulations do not constitute a highly diverse class of excipients, as the most extensively studied and applied compound is glutathione. From a biochemical perspective, glutathione (γ-glutamyl-L-cysteinylglycine, GSH) is the most abundant non-protein thiol in living organisms, particularly in eukaryotic cells. Over 90% occurs in its reduced form (GSH), while the oxidized form (GSSG) is generated upon oxidation and can be converted back to GSH by glutathione reductase. The strong electron-donating capacity of GSH, together with its relatively high intracellular levels (often reaching the millimolar range), helps sustain a reducing environment within cells. This property makes GSH a key antioxidant, safeguarding DNA, proteins, and other biomolecules from oxidative damage caused by reactive oxygen species and related agents [36].

GSH is present in at least two approved therapeutic protein lyophilized formulations, the recombinant Factor VIII product Advate^®^ as well as recombinant PEGylated Factor VIII product Adynovi^®^, demonstrating its feasibility as a functional excipient in commercial biologics [37,38,39]. In particular, Jameel et al. showed that a beneficial effect of GSH was observed at concentrations as low as 0.05 mg/mL, with subsequent formulations typically containing 0.2 mg/mL GSH [40]. Experimental work has further demonstrated its activity against peroxide-induced degradation: Knepp et al. showed that thiol antioxidants (cysteine, GSH, thioglycerol) were most effective in protecting recombinant growth factors from polysorbate-related oxidation, acting as chain-breaking antioxidants rather than through covalent adduct formation [41]. Similarly, Mahler et al. reported its role in reducing polysorbate degradation under oxidative stress [42]. However, its protective effect is not universal. In IL-2 formulations, GSH inhibited peroxide-driven oxidation post-lyophilization but was partially consumed by surfactant degradation products and failed to prevent oxidation during freeze-drying [43]. In other cases, GSH even accelerated degradation, likely due to disulfide scrambling [44,45]. Comparative studies of granulocyte colony-stimulating factor (G-CSF) formulations also suggest pH-dependent performance, with GSH showing limited efficacy in acidic formulations compared to methionine, but becoming more reactive under alkaline conditions [46].

Collectively, these findings highlight both the promise and limitations of GSH: while established as a functional excipient in marketed biologics, its stabilizing efficiency varies with protein, formulation conditions, and concentration. A summary of its reported uses is provided in Table 3.

## 4. Viscosity Reducers

High-concentration (HC) protein formulations, typically containing antibodies in the range of 100–200 mg/mL, have become essential for subcutaneous (SC) administration of biotherapeutics. This route offers significant advantages, allowing patients to self-administer effective doses at home without the need for hospitalization. However, such concentrated formulations present two critical challenges: (i) an increased tendency for protein aggregation and (ii) markedly elevated viscosity. Both issues pose risks to the stability, manufacturability, and deliverability of the final drug product. Viscosity is a particularly critical attribute in the development of such products. Protein solutions display an exponential rise in viscosity as concentration increases, primarily due to reversible self-association mediated by short-range protein–protein interactions. For instance, monoclonal antibody solutions at 125 mg/mL can become up to 60-fold more viscous than buffer alone, far exceeding the pharmaceutically acceptable limit of ~20 cP for SC injection [47]. Elevated viscosity not only complicates manufacturing and reconstitution of lyophilized products but also directly impacts syringeability, injection force, and patient comfort. In practice, high viscosity often necessitates larger-gauge needles, which in turn increases injection pain and reduces patient compliance. To mitigate these problems, excipients that weaken protein–protein interactions are frequently employed. By reducing self-association, such additives lower viscosity while simultaneously enhancing colloidal stability [10,48,49]. Among the most widely studied are amino acids and their salts, including arginine, lysine, proline and histidine, as well as common salts such as sodium chloride, sodium sulfate, and sodium acetate. Arginine, in particular, has been extensively investigated and used in multiple commercial biotherapeutic formulations. Its utility is twofold: it not only lowers viscosity in high-concentration solutions but also acts as a stabilizer during protein refolding and a general inhibitor of aggregation [10,14,21]. Similarly, proline has been shown to decrease viscosity as well as increase the colloidal stability at pH 6 of mAb formulation. The mechanism by which these amino acids exert their effects is thought to involve disruption of reversible protein–protein interactions, often by shielding surface charges, CH-π interactions or modulating hydration layers around the protein [50].

Short peptides are an appealing direction in the search for viscosity-reducing excipients because they expand the chemical space beyond single amino acids, offering a far greater diversity of structures and interaction motifs. Their modular nature enables fine-tuning of charge, hydrophobicity, and aromatic interactions, which can selectively modulate protein–protein interactions responsible for high viscosity in concentrated formulations. In a systematic screen, Proj et al. generated a 400-dipeptide library and selected candidates with at least one pKa between 5 and 7. Eleven dipeptides containing three or more charged groups reduced viscosity, supporting the utility of multi-charged sequences as dual excipients (viscosity reduction + buffering) [51]. Building on these results, Prašnikar et al. further explored chemical spaces suggested in earlier studies. Proline-containing dipeptides were particularly effective in terms of viscosity reduction (mean relative viscosity ≈ 0.70), consistent with additional π-, hydrogen-bonding, and hydrophobic contributions [52]. Among them, Pro-Pro stood out, lowering viscosity in both aqueous and formulated mAb solutions (protein concentration of 170 mg/mL) below the critical 20 cP threshold at ≥50 mM—something proline could not achieve even at 200 mM. Interestingly, Pro–Pro exhibited a biphasic, concentration-dependent effect on viscosity reduction. The maximal viscosity reduction was observed at 100 mM, resembling behaviors previously reported for glycine, lysine, and sodium chloride. Accelerated stability studies further indicated enhanced physical stability of dipeptide-based formulations (Pro–Pro, Pro–Gln, Pro–Tyr, Gly–Pro) [53]. While proline derivatives dominated academic studies, patents have mostly explored arginine-based peptides, building on the well-established viscosity-reducing properties of the parent amino acid. For example, Soane et al. reported the largest viscosity decrease in a 280 mg/mL bovine gamma globulin formulation for Arg–Glu at 165 mM, although higher concentrations (330 mM) reversed the effect and increased viscosity [54]. Gu et al. described a similar threshold behavior in tezepelumab formulations, further underscoring the concentration-dependent nature of peptide excipients [55]. Beyond mixed dipeptides, Bowen et al. showed that arginine homopeptides such as Arg–Arg and Arg–Arg–Arg were more effective viscosity reducers than simple arginine hydrochloride [56]. In a complementary approach, Agrawal et al. reported that N-acetylated dipeptides matched the viscosity-lowering effect of N-acetyl-arginine but offered much higher solubility, allowing their use at elevated concentrations and achieving greater overall reductions [57]. Taken together, these studies outline a clear progression—from mixed dipeptides, through homopeptides, to chemically modified derivatives— illustrating the versatile design space of peptides as next-generation viscosity-modifying excipients with clear opportunities for rational optimization. Short peptide excipients evaluated in these studies are summarized in Table 4.

## 5. Excipients for Lyophilizates

Lyophilization is the method of choice for stabilizing therapeutic proteins that are otherwise insufficiently stable in aqueous solution. Although highly effective, freeze-drying is a time- and energy-demanding process, with the primary drying phase typically being the major bottleneck. To obtain stable products while also ensuring process efficiency, optimization must be approached at two levels: careful design of process parameters and rational selection of excipients. Excipients are essential not only for preserving protein stability during freezing and drying, but also for ensuring critical quality attributes of the final product, such as cake appearance, reconstitution time, and residual water content. Moreover, excipients can significantly influence the overall efficiency of the freeze-drying cycle, thereby affecting production costs and scalability. Cryoprotectants are excipients that stabilize protein structure during freezing by mitigating ice crystal damage and phase separation, while lyoprotectants act during drying, embedding the protein in an amorphous glassy matrix that restricts mobility and prevents unfolding or aggregation. Disaccharides such as sucrose and trehalose are the most frequently used examples, whereas amino acids such as arginine or glycine may also contribute under certain conditions. Protein stabilization in the amorphous phase has been explained by two complementary concepts: the water-replacement hypothesis, where excipients substitute for water molecules via hydrogen bonding, and the vitrification hypothesis, where the glassy state mechanically immobilizes the protein. Both mechanisms are influenced by the glass transition temperature (Tg) of the system, which must remain well above the intended storage temperature to ensure long-term protection. Equally important are bulking agents, whose function is to provide mechanical strength and elegant appearance to the lyophilized cake, while enabling efficient sublimation by creating porous structures. Mannitol and glycine are the most widely used crystalline bulking agents, valued for their ability to crystallize during freezing and thereby yield robust cakes with relatively high collapse or eutectic temperatures. This allows more aggressive primary drying conditions and shorter cycles. However, crystallization must be complete; otherwise, residual amorphous fractions pose a risk of lyophilizate collapse. In contrast, amorphous excipients such as sucrose or trehalose can serve simultaneously as stabilizers and bulking agents, but their low collapse temperatures limit their usefulness for cycle optimization. Despite their established roles, the selection of cryoprotectants, lyoprotectants, and bulking agents remains challenging. The typical toolbox of excipients for freeze-drying is surprisingly narrow given the enormous diversity of therapeutic proteins. It is unlikely that this limited set can always provide optimal stabilization. Amino acids, for example, are generally not suitable as primary lyoprotectants or bulking agents at neutral pH, apart from glycine as a bulking agent. They are more often explored as secondary protectants in combination with carbohydrates. Nevertheless, issues such as low solubility at neutral pH and a tendency to crystallize under freeze-drying conditions restrict their broader application. The latter point is critical: stabilizers must remain amorphous to function effectively, since crystallization prevents the close molecular interactions required for protein protection. Conversely, the bulking agent is expected to crystallize, to prevent cake collapse and ensure structural robustness, underscoring the need for complementary functions of different excipients within a formulation. Finally, the physicochemical properties of excipients—particularly molecular weight, glass transition behavior (Tg′ of maximally freeze-concentrated solutes and Tg of the dried amorphous matrix), and crystallization tendency—are decisive factors that determine both physical stability and manufacturability. A rationally chosen combination of amorphous lyoprotectants and crystalline bulking agents remains the foundation of current formulation practice, but the limitations of the existing excipient palette highlight the need for exploring new molecules that may broaden the design space for lyophilized protein therapeutics [11,58,59,60].

In this landscape, short peptides may constitute as a novel class of multifunctional excipients for lyophilized formulations. Specifically, short peptides, similar to proteins, can exhibit relatively high glass transition temperatures (Tg′) as well as provide both cryoprotective and lyoprotective effects during lyophilization [61,62]. At the same time, they are less costly to produce than larger proteins and avoid many of the formulation complexities that arise when using protein-based excipients, such as potential immunogenicity, structural heterogeneity, or interference in analytical assays. For example, the model tripeptide FK906 has been reported to display a Tg′ of approximately −19 °C, which is substantially higher than that of sucrose (−32 °C), suggesting that short peptides could support more robust lyophilization processes [63]. However, apart from FK906, there is an almost complete absence of quantitative Tg′ values for short peptides in the literature. Most systematic investigations of freeze-concentrated glass transition behavior have focused instead on sugars, polymers, or proteins. This clear knowledge gap highlights the need for more systematic evaluation of di- and tripeptides in the context of lyophilized formulations. To date, Gly-Gly remains the only short peptide explicitly described as an excipient in lyophilized protein formulations, making it the natural starting point for further discussion.

Hansen (Novo Nordisk) in his patent literature presented that Gly-Gly may serve a dual role in freeze-drying, functioning as a bulking agent that provides structural integrity to the lyophilized cake while simultaneously acting as a stabilizer against protein aggregation. In addition, glycylglycine can contribute buffering capacity in solution prior to lyophilization, thereby reducing the overall number of excipients required in the final formulation. Specifically the Author using Gly-Gly as a sole bulking agent (with optimal range of concentration of 25–40 mg/mL) resulted in elegant cake with improved stability. Combination of Gly-Gly with sucrose resulted in both more elegant cake as well as superior physical and chemical stability of modified recombinant factor VIIa. However, the patent does not provide data on critical thermal parameters of the formulation, such as glass transition or collapse temperatures, nor does it clarify whether Gly-Gly forms an amorphous or crystalline matrix upon lyophilization [64]. In further patents by Novo Nordisk, Jensen et al. and Hansen et al. also described usage of Gly-Gly in recombinant factor VIIa formulation, but this time only as buffering agent (up to 1.5 mg/mL) [65,66]. Furthermore, in patent by Hile et al., formulations of bone morphogenetic protein 7 containing Gly-Gly buffer (10 mM) exhibited a lower aggregation propensity than those buffered with lactate (10 mM) [67]. Lastly, is an excipient in the approved rFVIIa product NovoSeven^®^/NovoSeven^®^ RT [68,69].

## 6. Conclusions and Future Perspectives

Formulating protein therapeutics remains a complex task due to the intrinsic instability of proteins and the limited repertoire of excipients available to mitigate these challenges. Excipients, though essential to the performance of pharmaceutical products, often receive far less attention and investment compared to the discovery of novel drugs. Yet, they function as the infrastructure of the pharmaceutical sector, and their absence or limitations can directly hinder innovation, delay development timelines, or even prevent new therapies from reaching patients. Industry surveys confirm that the restricted pool of recognized excipients frequently forces formulators to rely on suboptimal solutions, underscoring the urgent need for innovation in this area [70].

Short peptides represent a promising approach to expand the excipient toolbox. They combine high chemical diversity, biocompatibility, and tunable physicochemical properties, enabling them to act as stabilizers, viscosity modifiers, antioxidants, cryoprotectants, or bulking agents in protein formulations. Furthermore, their structural modularity allows fine-tuning of molecular mass, charge distribution, hydrophobicity, and hydrogen-bonding capacity, features that can translate into unique stabilization mechanisms. Importantly, peptides may act dually as stabilizers and bulking agents, protecting proteins against conformational stress while simultaneously contributing to cake structure. As an example, Arg-Phe may act both as a stabilizer and a viscosity-reducing agent, whereas Gly-Gly can function as a bulking agent, lyoprotectant/cryoprotectant, and buffer [26,55,64]. Moreover, feasibility of short peptide excipients is not only theoretical—glutathione is already present in approved protein products such as Advate^®^ and Adynovi^®^, while Gly-Gly is a functional excipient in the marketed rFVIIa formulation NovoSeven^®^ [38,39,68]. These examples demonstrate that peptide-based excipients can be translated into commercial biologics, paving the way for broader systematic exploration of di- and tripeptides as next-generation formulation components.

**Figure 2:**
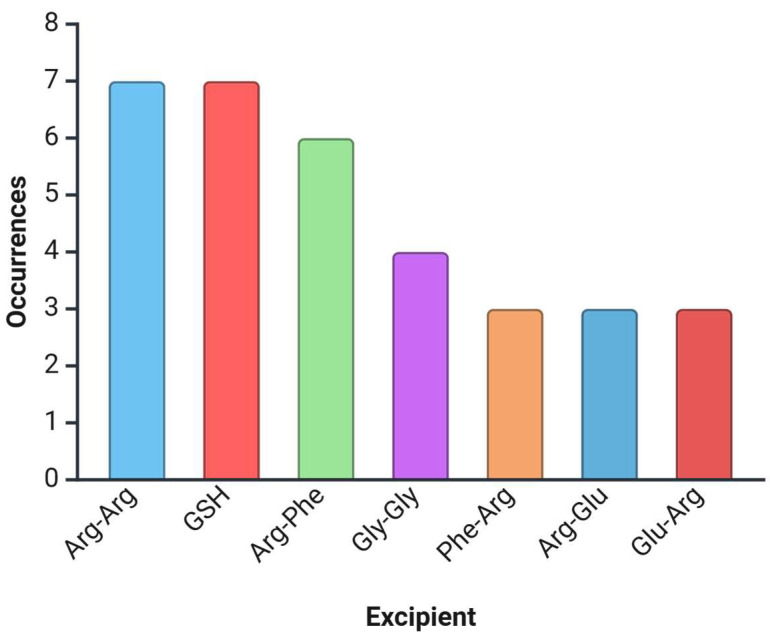
Frequency of appearances of individual peptide excipients in the scientific and patent literature.

A survey of the available literature and patent landscape indicates that certain short peptides are investigated far more frequently than others, reflecting both their functional promise and ease of incorporation into protein formulations. In particular, arginine-based homopeptides (e.g., Arg–Arg) and mixed dipeptides (such as Arg–Phe, Phe–Arg, Arg-Glu and Glu-Arg) dominate reported studies, while GSH and Gly–Gly remain the only peptides consistently reported as an antioxidant and as a lyophilization excipient, respectively. To highlight these trends, Figure 2 summarizes the distribution of peptides most often explored, and Table 5 presents the four most frequently reported candidates, together with their described functional roles in protein stabilization and formulation.

Despite their potential, several challenges must be addressed before short peptides can be more broadly adopted as excipients. The cost and scalability of peptide production remain limiting factors compared to conventional excipients. Nevertheless, the peptide manufacturing sector has grown into a robust and dynamic industry. Advances in chemical synthesis, recombinant expression, and enzymatic methods have greatly expanded the chemical space of peptides, enabling access to more complex and diverse sequences. While peptides are relatively straightforward to generate on a small scale—particularly with solid-phase peptide synthesis (SPPS), which is highly automatable and allows rapid production of multiple candidates—large-scale manufacturing is considerably more complex. Production routes depend strongly on the target sequence, chosen modifications, and quality requirements. Recombinant expression is often slower to establish and cost-intensive at the outset, but it becomes competitive at scale and is generally viewed as a more environmentally sustainable option. In contrast, chemical synthesis offers speed and flexibility in the early development phase and is compatible with noncanonical amino acids or special sequence motifs, yet it relies heavily on organic solvents, protecting groups, and auxiliary chemicals, making it less ‘green’ than recombinant processes. Despite these challenges, steady improvements in synthesis, purification, and isolation technologies have reduced average costs—by nearly 50% over the last three decades—and have driven the continued expansion of the peptide manufacturing industry. With more than 70 approved peptide-based products and over 400 currently in clinical development, the coming years are expected to bring further opportunities for innovation, including in the context of peptide-based excipients [71,72,73]. In addition to manufacturing considerations, short peptides composed of natural amino acids exhibit a favorable safety profile, characterized by low cytotoxicity in vitro, absence of acute systemic toxicity in vivo, minimal genotoxic or sensitization effects, and generally low immunogenic potential, especially when manufactured at high purity without unnatural modifications. Their metabolites are amino acids, which further reduces the likelihood of long-term accumulation or toxic effects. Nevertheless, the available data are fragmented and often derived from therapeutic or bioactive peptides rather than from those specifically applied as excipients in protein formulations. Systematic studies addressing long-term safety and immunogenicity under clinically relevant conditions are still lacking, leaving an important knowledge gap to be addressed in future research [13,74,75]. Translating these favorable properties into regulatory acceptance is not straightforward. According to guidelines and definitions, excipients used for the first time in medicinal products are classified as novel [76,77,78]. In the United States, regulatory precedence is established through inclusion in the Inactive Ingredient Database (IID), which lists excipients together with their route, dosage form, and maximum approved level. However, excipients used in non-vaccine biologics (including therapeutic proteins) licensed through the BLA pathway may not appear in the IID. As an example, GSH is employed in the antihemophilic factor ADVATE^®^ despite being absent from this database [70]. For novel excipients, manufacturers must provide extensive safety packages, and the amount of data required depends on the degree of novelty and intended use. A practical illustration is provided by Gly-Gly in NovoSeven^®^, where the applicant submitted acute, repeat-dose, reproduction, and mutagenicity studies [79]. This additional regulatory burden, combined with limited financial incentives, may explain why no marketed biologics currently include short peptide excipients beyond GSH and Gly-Gly, despite their promising functionality. On the other hand, recent initiatives suggest a more supportive environment is emerging. In 2021, the US FDA launched the voluntary *Novel Excipient Review Pilot Program*, enabling manufacturers to seek regulatory feedback on new excipients prior to their use in drug products [80]. Additionally, the COVID-19 pandemic demonstrated that accelerated timelines are possible, as the development of mRNA vaccines relied on novel lipid excipients that reached the market in under two years [81]. These developments may help pave the way for new excipients, including short peptides, to advance into clinical use and, ultimately, commercial therapeutic protein formulations. Finally, the choice of counterion can critically influence solubility, stability, and overall formulation performance, underscoring the need for deliberate selection and systematic evaluation during excipient development [82]. For short peptides with a net positive charge, this issue is particularly relevant, yet it has not been extensively investigated in the literature. The limited evidence available comes from studies such as that by Shukla et al., who examined chloride and sulfate salts of polyarginine (n = 1–4) and demonstrated that the nature of the counterion can markedly affect the stability of model proteins such as α-chymotrypsinogen A and concanavalin A [28]. These observations suggest that counterion identity may be a decisive factor in the formulation behavior of peptide excipients, warranting broader exploration in future studies.

Overall, short peptides represent a promising class of excipients with the potential to address key unmet needs in protein stabilization. Further focused research in this area may enable the development of more robust, efficient, and patient-friendly formulations.

## Figures and Tables

**Table 1:** Excipients utilized in parenteral protein formulations [12]. Several excipients have dual or multiple roles and, therefore, appear in more than one category.

Excipient Category	Function	Examples in Approved Products
Buffering Agents	Stabilize the pH of the solution	Histidine Buffer Citrate Buffer Phosphate Buffer
Tonicity Modifiers	Adjust tonicity and ionic strength (in case of ionic excipients)	NaCl Sucrose Trehalose Mannitol Sorbitol
Surfactants	Inhibit protein adsorption (competitive) and surface-induced protein denaturation	Polysorbate 20 Polysorbate 80 Poloxamer 188
Stabilizers	Enhance both the conformational and colloidal stability of proteins	Sucrose Trehalose Arginine Proline Sorbitol
Antioxidants	Inhibit oxidation	Methionine
Viscosity Modifiers	Reduce solution viscosity	Arginine Proline Glycine NaCl
Bulking Agents	Provide the appropriate structure and appearance of the lyophilizate and optimize its collapse temperature	Glycine Mannitol Sucrose Trehalose
Lyoprotectants	Enhance stability of proteins during exposure to freeze-drying-related stresses	Sucrose Trehalose Arginine

**Table 2:** List of short peptide stabilizers studied in articles cited in this review.

Protein	Peptide Stabilizer	Impact	Reference
cytochrome c	Ala-His	No pronounced effect	[22]
	Phe-Gly	Aggregation inhibition	
	Gly-Phe	No pronounced effect	
alcohol dehydrogenase	Arg-Phe	Aggregation enhancement (1 mM) Aggregation inhibition (4.5 mM)	[25]
	Ala-Phe-Lys	Aggregation enhancement (1 mM) Aggregation inhibition (4.5 mM)	
α-lactalbumin	Arg-Phe	Aggregation enhancement	[24]
	Glu-Val-Phe	No pronounced effect	
	Asp-Phe	No pronounced effect	
alcohol dehydrogenase	Arg-Phe	Aggregation enhancement (1 mM, 38–39 °C) No pronounced effect (1 mM, 40–42 °C)	[23]
	Asp-Phe	Aggregation enhancement	
insulin	Arg-Arg	Aggregation enhancement (pH 7.5) Aggregation inhibition (pH 3.7 and 5.5)	[26]
	Arg-Phe	Aggregation inhibition	
	Leu-Arg	Aggregation inhibition (100 mM, pH 5.5)	
	Arg-Glu		
	Phe-Arg		
	Glu-Arg		
	Arg-Val	No pronounced effect	
	Val-Arg		
	Glu-Arg-NH_2_		
	Arg-Phe-NH_2_		
	Arg-Glu-NH_2_		
recombinant human serum albumin	Arg-Arg	Aggregation enhancement	[27]
	Arg-Phe		
	Phe-Arg		
	Glu-Glu		
	Arg-Glu		
	Glu-Arg		
ovalbumin	Arg-Arg	Aggregation enhancement	
	Arg-Phe		
	Phe-Arg		
	Glu-Glu		
	Arg-Glu		
	Glu-Arg		
α-chymotrypsinogen A	Arg-Arg	No pronounced effect	[27]
	Arg-Phe		
	Phe-Arg	Aggregation inhibition	
	Glu-Glu		
	Arg-Glu		
	Glu-Arg		
α-chymotrypsinogen A, concanavalin A	Arg-Arg	Aggregation inhibition (sulfate salts, chloride salts at concentration of 0.1 M) Aggregation enhancement (chloride salts at concentration of 0.25 M)	[28]
	Arg-Arg-Arg		
	Arg-Arg-Arg-Arg		
interferon-α2a	Gly-D-Asn	Aggregation inhibition	[29,30]
MEDI-578	Ala-Pro	No pronounced effect	[31]
	Gly-Gly		
	Gly-Gln		
	His-His	Attractive self-interactions reduction	
	His-Lys		
	His-Ser		
	Arg-Arg		
motavizumab	Ala-Pro	No pronounced effect	
	Gly-Gly	No pronounced effect	
	Gly-Gln	Repulsive interactions reduction	
	His-His	Repulsive interactions enhancement	
	His-Lys	No pronounced effect	
	His-Ser	No pronounced effect	
	Arg-Arg	No pronounced effect	
erythropoietin omega	Gly-Gly	Protein loss reduction	[32]
	Gly-Gly-Gly		
	Gly-Tyr		
	Gly-Phe		
	Gly-His		
	Gly-Asp		
	Gly-Ala		
	Ala-Gly		
	Ala-Ala		

**Table 3:** Impact of GSH excipient studied in articles cited in this review.

Protein	GSH Concentration	Impact	Reference
recombinant factor VIII	16 mM	Prevent oxidation ^1^	[40]
recombinant human Ciliary Neurotrophic Factor	15 mM	Prevent peroxide-induced oxidation	[41]
recombinant human Nerve Growth Factor			
N/A ^2^	3 mM	Minimize polysorbate degradation	[42]
IL-2 mutein	16 mM	Partially prevents peroxide-induced oxidation	[43]
rhIL-2A125	0.3 mM	Accelerate degradation of the protein	[44]
otelixizumab	5 mM	Accelerate degradation of the protein	[45]
G-CSF	60 mM	No obvious effect (pH 4.5)	[46]

^1^ GSH is used as an excipient in approved recombinant factor VIII as well as recombinant PEGylated Factor VIII formulations (Advate^®^ and Adynovate^®^) [37,38,39]; ^2^ Formulation prepared in the absence of protein (“placebo”).

**Table 4:** List of short peptide viscosity reducers studied in articles cited in this review.

Protein (Concentration)	Peptide Viscosity Reducer	Viscosity Reduction (Excipient Concentration)	Reference
mAb A (150 mg/mL), mAb B (150 mg/mL)	His-Tyr	mAb A: −70% (25 mM) mAb B: −56% (25 mM)	[51]
	His-Ala	mAb A: −65% (25 mM) mAb B: −43% (25 mM)	
	His-Gly	mAb A: −66% (25 mM) mAb B: −40% (25 mM)	
	His-Ser	mAb A: −61% (25 mM) mAb B: −42% (25 mM)	
	His-Phe	mAb A: −60% (25 mM) mAb B: −43% (25 mM)	
	His-Lys	mAb A: −58% (25 mM) mAb B: −43% (25 mM)	
	His-Arg	mAb A: −58% (25 mM) mAb B: −39% (25 mM)	
	His-Val	mAb A: −43% (25 mM) mAb B: −9% (25 mM)	
	Asp-Gly	mAb A: −54% (25 mM) mAb B: −35% (25 mM)	
	His-Asp	mAb A: −57% (25 mM) mAb B: −36% (25 mM)	
	Asp-Leu	mAb A: −53% (25 mM) mAb B: −26% (25 mM)	
mAb (150 mg/mL)	Pro-Pro	−51% (25 mM)	[52]
	Pro-Gln	−31% (25 mM)	
	Pro-Tyr	−37% (25 mM)	
	Pro-Ala	−22% (25 mM)	
	Gly-Pro	−34% (25 mM)	
	Pro-Gly	−9% (25 mM)	
	Val-Pro	−26% (25 mM)	
	Val-Pro-Pro	−31% (25 mM)	
	Gly-Pro-Ala	−12%(25 mM)	
mAb (170 mg/mL)	Pro-Pro	−49% (25 mM) −48% (50 mM) −59% (100 mM) −33% (200 mM)	[53]
	Pro-Gln	−35% (25 mM)	
	Pro-Tyr	−49% (25 mM)	
	Gly-Pro	−40% (25 mM)	
	Val-Pro	−33% (25 mM)	
	Gly-Pro-Ala	−52% (25 mM)	
BGG (280 mg/mL)	Arg-Arg-Arg-Arg-Arg	−32% (50 mM) −30% (100 mM)	[54]
	His-His-His-His-His	−24% (25 mM) −35% (50 mM) −22% (100 mM)	
	Trp-Trp-Lys-Lys-Lys	−24% (50 mM) −25% (100 mM)	
	Asp-Asp-Asp-Asp-Asp	+4% (50 mM) +29% (100 mM)	
	Leu-Glu	−9% (50 mM) +10% (100 mM)	
	Tyr-Glu	−30% (50 mM) −14% (100 mM)	
	Arg-Pro	−35% (50 mM) −33% (100 mM)	
	Arg-Lys	−33% (50 mM) −19% (100 mM)	
	Arg-His	−34% (50 mM) −9% (100 mM)	
	Arg-Arg	−28% (50 mM) −22% (100 mM)	
	Arg-Glu	−37% (50 mM) −16% (100 mM)	
tezepelumab (128 mg/mL)	Arg-Arg-Arg-Arg	−53% (100 mM)	[55]
	Arg-Arg	−46% (100 mM) −49% (200 mM)	
	Arg-Lys	−46% (100 mM) −47% (200 mM)	
	Arg-Phe	−7% (10 mM) −44% (100 mM) −50% (200 mM) −43% (500 mM)	
	Arg-Pro	−32% (200 mM)	
	Arg-Val	−51% (200 mM)	
	Arg-Ala	−43% (100 mM) −47% (200 mM)	
	Asp-Arg	−34% (200 mM)	
	Lys-Arg	−11% (10 mM) −52% (200 mM) −36% (500 mM)	
	Pro-Arg	0% (10 mM) −35% (100 mM) −46% (200 mM) −49% (500 mM)	
	Leu-Arg	−49% (200 mM)	
	Val-Arg	−47% (200 mM)	
	Ala-Arg	−47% (200 mM)	
evolocumab (150 mg/mL)	Arg-Arg	−68% (150 mM)	[55]
	Arg-Phe	−73% (150 mM)	
	Arg-Pro	−65% (150 mM)	
	Arg-Tyr	−67% (150 mM)	
	Arg-Ala	−62% (150 mM)	
	Arg-Val	−61% (150 mM)	
	Phe-Arg	−75% (150 mM)	
	Pro-Arg	−75% (150 mM)	
	Val-Arg	−70% (150 mM)	
	Ala-Arg	−68% (150 mM)	
anti-CD4 mAb (222–244 mg/mL)	Arg-Arg	−74% (30 mM) −72% (150 mM)	[56] ^1^
	Arg-Arg-Arg	−80% (30 mM) −82% (150 mM)	
Ab1 (210 mg/mL) Ab2 (210 mg/mL) Ab3 (210 mg/mL)	Ac-Pro-Arg	Ab1: −87% (150 mM) Ab2: −77% (150 mM) Ab3: −34% (150 mM)	[57] ^1^
	Ac-Ser-Arg	Ab1: −84% (150 mM) Ab2: −63% (150 mM) Ab3: −27% (150 mM)	
	Glu-Arg	Ab1: −56% (25 mM) Ab2: −53% (25 mM) Ab3: −15% (25 mM)	
	Ac-Pro-Arg-NH_2_	Ab1: −83% (150 mM) Ab2: −88% (150 mM) Ab3: −46% (150 mM)	

^1^ The viscosity reduction was calculated based on the data available in the patent literature.

**Table 5:** Details on most frequently reported short peptide excipients in the scientific and patent literature.

Peptide Excipient	Concentration Range	Comment
Arg-Arg	20–250 mM	Arg-Arg can act both as stabilizer and viscosity reducing agent. The boundary between aggregation inhibition and enhancement may be related to protein type and charge, formulation pH, excipient concentration and peptide counterion.
GSH	0.3–60 mM	GSH is present in at least two approved therapeutic protein lyophilized formulations. Its antioxidant activity is dependent on protein type, excipient concentration and formulation pH. It can potentially induce a disulfide shuffling.
Arg-Phe	0.5–500 mM	Arg-Phe can act both as stabilizer and viscosity reducing agent. The boundary between aggregation inhibition and enhancement may be related to protein type and charge, formulation pH, excipient concentration and incubation temperature. Exceeding a certain concentration threshold (200 mM) can lead to a reversal of the viscosity reducing effect.
Gly-Gly	10–300 mM	Gly-Gly is present in at least two approved therapeutic protein lyophilized formulations. Its usage as buffering agent (approx. 10 mM) as well as cryo/lyoprotectant and bulking agent (approx. 300 mM) has been demonstrated in patent literature. However, no specific mechanism underlying its stabilizing effect in lyophilized formulations has been reported to date.

## Data Availability

Data is contained within the article.

## Abbreviations

The following abbreviations are used in this manuscript:

Ab	antibody
Ala	alanine
Arg	arginine
Asn	asparagine
Asp	aspartic acid
BGG	bovine gamma globulin
BLA	biologics license application
Cys	cysteine
EPO	erythropoietin
(US) FDA	the united states food and drug administration
G-CSF	granulocyte colony-stimulating factor
Gln	glutamine
Glu	glutamic acid
Gly	glycine
GSH	glutathione (reduced form)
GSSH	glutathione (oxidized form)
HC	high-concentration
His	histidine
hPTH	human parathormone
HSA	human serum albumin
IID	inactive ingredient database
Ile	isoleucine
Leu	leucine
Lys	lysine
mAb	monoclonal antibody
Met	methionine
Phe	phenylalanine
Pro	proline
rAHF	granulocyte colony-stimulating factor
rhIL-2	recombinant human interleukin-2
rHSA	recombinant human serum albumin
ROS	reactive oxygen species
SC	subcutaneous
Ser	serine
SPPS	solid-phase peptide synthesis
Tg	glass transition temperature of the dried amorphous matrix
Tg’	glass transition temperature of maximally freeze-concentrated solutes
Thr	threonine
Trp	tryptophan
Tyr	tyrosine
Val	valine
